# A Single Ascending-Dose Study of the Safety, Tolerability, Pharmacokinetics, and Pharmacodynamics of the Novel Respiratory Stimulant ENA-001

**DOI:** 10.7759/cureus.55057

**Published:** 2024-02-27

**Authors:** Joseph Pergolizzi, Thomas L Miller, Jeanette Mathews, Robert B Raffa, Robert Colucci, Frank J Diana, Errol Gould

**Affiliations:** 1 Anesthesiology, Enalare Therapeutics, Inc., Princeton, USA; 2 Clinical Development, Enalare Therapeutics, Inc., Princeton, USA; 3 Research and Development Operations, Enalare Therapeutics, Inc., Princeton, USA; 4 School of Pharmacy, Temple University (Emeritus), Philadelphia, USA; 5 Pharmacology, NEMA Research, Inc., Naples, USA; 6 Pulmonology, Enalare Therapeutics, Inc., Princeton, USA; 7 Medical Affairs, Enalare Therapeutics, Inc., Princeton, USA

**Keywords:** respiratory stimulant, respiratory depression, postoperative respiratory depression, minute ventilation, gal-021, ena-001

## Abstract

Background

ENA-001 (formerly known as GAL-021) is a novel, first-in-class respiratory stimulant. Pharmacokinetic and pharmacodynamic properties, plus safety and tolerability, were assessed in a randomized, single-center study of healthy volunteers.

Methodology

This four-period study was designed to test continuous two-hour intravenous infusion regimens of ENA-001 at doses of 0.96, 1.44, and 1.92 mg/kg/hour versus placebo. Each participant received four infusions with a seven-day minimum washout between them: one infusion each of the three doses of ENA-001 and one placebo. Pharmacokinetic and pharmacodynamic parameters were assessed and adverse events were recorded.

Results

A total of 17 participants completed the study. ENA-001 was generally safe and well tolerated over the dose range studied (0.96 to 1.92 mg/kg/hour). ENA-001 was able to drive hyperventilation in a dose-dependent manner in healthy participants. Increases in ventilation due to ENA-001 were not associated with like-magnitude blood pressure response. ENA-001-stimulated decreases in ETCO_2_ were associated with small, statistically significant, increases in SpO_2_ levels. Hyperventilation occurred in two participants at the highest dose level, leading to study discontinuation. The terminal half-life of ENA-001 was 6.33 hours.

Conclusions

The respiratory stimulant ENA-001 demonstrated well-behaved pharmacokinetics following the two-hour infusion. Mean peak plasma concentrations and the mean total systemic exposure values were approximately dose-proportional in the dose range studied.

## Introduction

Ventilatory insufficiency is a potentially life-threatening in-hospital complication that is associated with increased morbidity as well as greater utilization of resources and higher costs [1-3]. Respiratory failure in hospitalized patients is increasing in America; it has gone up by 83% from 2002 to 2017 [3]. Postoperative respiratory depression remains an underestimated condition that is challenging to treat. Postoperative respiratory depression is the result of a cascade of physiologic processes in which the patient fails to overcome the pharmacologic effects of anesthesia and perioperative opioids, impairing the resumption of normal breathing [4]. Despite its prevalence and potentially life-threatening consequences, postoperative respiratory depression is not fully elucidated [5].

Some risk factors for postoperative respiratory depression mirror the changing American demographic profile: older age (≥60 years), obesity, obstructive sleep apnea, and cardiovascular disease such as congestive heart failure [6,7]. Other risk factors include smoking and hypoalbuminemia, which may be exacerbated by the postoperative administration of opioid analgesics [8]. A recent review suggests that all surgical patients are susceptible to deleterious respiratory effects after surgery under general anesthesia, and these adverse respiratory effects may occur after hospital discharge [9].

In a healthy individual, respiration is controlled by the brainstem with inputs from the cortex and peripheral nervous system. Chemoreceptors in the brainstem and peripheral nerves respond to oxygen tension, carbon dioxide tension, and chemical stimuli, such as pH. Type 1 glomus cells are located in the carotid body at the point where the internal and external carotid arteries bifurcate; these type 1 glomus cells act as the main type of hypoxia sensor in the periphery [10]. The carotid bodies transmit impulses along the carotid sinus nerve to the brainstem region, such as the nucleus tractus solitarius, a primary site where ventilatory sensory afferents from the lungs, airways, and peripheral chemoreceptors converge. The nucleus tractus solitarius connects to the pre-Bötzinger complex, thought to be the pacemaker for natural respiration [10]. It has been observed that the inhibition of certain ion channels in the glomus cells of the carotid body can stimulate ventilation [11].

The novel agent examined in this study, ENA-001 (Enalare Therapeutics, Inc., Princeton, New Jersey, USA), previously GAL-021, is a first-in-class, fast-acting, intravenous (IV) agent. ENA-001 inhibits the voltage-activated “big” potassium (BK) channels located in the carotid body [12], and in so doing stimulates respiration and increases minute ventilation by increasing tidal volume and inducing a modest increase in ventilatory rate.

This study aimed to evaluate the safety, tolerability, pharmacokinetic (PK), and pharmacodynamic (PD) properties of ENA-001 at higher doses than those used in the first-in-human studies of ENA-001 [13]. This earlier double-blind randomized controlled trial with 30 participants consisted of IV infusions of between 0.1 and 0.96 mg/kg for one hour at the high dose level and intermediate doses for four hours [13]. The rationale behind this study was the use of higher doses. The secondary objective was to gather evidence about the agent’s bioavailability.

## Materials and methods

The study was designed as a four-period, randomized, double-blind, placebo-controlled, single-center study using an escalating single dose. The study was blinded for treatment but not for dose level. Investigators evaluated an ascending, repeated, single dose of ENA-001 versus placebo in healthy volunteers for safety and tolerability.

Participants were admitted to the study center the day before their first dose (Day 1 commenced with the first dose). Before dosing, safety and laboratory testing were conducted. Participants fasted overnight, and, in the morning, baseline assessments were done, including electrocardiography (ECG), blood pressure, and ventilatory monitoring. Participants then received a continuous IV infusion over two hours of the first dose (either the study drug or placebo). Safety, tolerability, PD assessments, and PK samples were taken for up to 12 hours afterward. The following morning, final assessments were done and the participants were discharged from the center.

On the first day of each dose level, three participants were treated; two received the study drug, and one the placebo. If no significant adverse events (AEs) occurred, an additional six participants were treated the next day, following the randomization schedule. For sentinel participants in dosing group 2 and thereafter, duplicate PK samples were collected at selected time points on Day 1 and analyzed no later than the next day. Thus, the PK information was available to the clinical team before the next higher dose increment was administered. Before this next dose was administered, a safety review was conducted using ECGs, blood pressure, AE reports, and PK parameters from at least two participants. Sentinel PK and analysis were not performed on dose level 1 (0.96 mg/kg/hour) because PK for this dose level has been well-defined [13]. Sentinel duplicate PK samples were evaluated as part of the dose-escalation criteria but these data were not included in the final PK analysis. There were three doses (0.96, 1.44, and 1.92 mg/kg/hour) and placebo. A washout period of at least seven days occurred between treatments.

PD endpoints were assessed using two independent systems: the Dräger Infinity patient monitoring system (Drägerwerk AG, Lübeck, Germany) and a portable NOX-T3 ventilatory monitoring system (Nox Medical, Reykjavík, Iceland). The Dräger Infinity system is an inpatient monitor that can measure minute ventilation, respiratory rate, and tidal volume using a pneumotachometer, and transcutaneous oxygen hemoglobin saturation; it also has a Capnostat sensor to measure end-tidal carbon dioxide levels. The NOX-T3 device is a portable home monitoring system designed for sleep apnea patients but used in this study for real-time measurement of respiratory rate, tidal volume via respiratory inductance plethysmography, transcutaneous oxygen hemoglobin saturation, actigraphy, and subject safety.

AEs were monitored and recorded by the investigator.

Participants were adults, aged 18 to 45 years, who provided written informed consent (Institutional Review Board ZNA/OCMA in Antwerp, Belgium, Protocol No. BE-80-1120316 and GAL-021-102, Ethics Committee approval #4002). This study was conducted at one site, the SGS Life Sciences Services in Antwerp, Belgium.

Inclusion criteria were a body mass index of 18 to 30 kg/m^2^, weight ≥60 kg and ≤90 kg, and normal values on clinical laboratory tests and physical examination, including ECG. Females had to be postmenopausal, surgically sterilized, or confirmed by testing to be not pregnant. Non-vasectomized males agreed to use a condom or abstain from sexual activity during the study and for at least three months after taking the final dose of study medication. Participants were excluded if they had a diagnosed psychiatric condition requiring daily medication, active substance use disorder or a history thereof, or had donated blood within 60 days of screening or plasma within seven days. Those with a history of dyspnea, asthma, tuberculosis, chronic obstructive pulmonary disease, sleep apnea, or other lung/pulmonary conditions were excluded. Participants with a history of malignancy, those who consumed large quantities of coffee or tea, and those considered by investigators to be unable to participate fully in the study were excluded.

The test drug in the study was ENA-001, prepared as a sterile product ready for dilution in normal saline for administration by continuous IV infusion. To mitigate possible injection-site reactions, it was diluted with normal saline and co-infused with Ringer’s lactate for all doses (~2:1 infusion rate of Ringer’s lactate to active) at a rate of 225 mL/hour. Dosing was based on the subject’s weight on a mg/kg basis. All doses were infused by continuous IV infusion over two hours. The matching placebo was a sterile normal saline solution co-infused with Ringer’s lactate at the same rate. Both the study drug and placebo were colorless liquids that looked similar.

Safety and tolerability were assessed using laboratory tests (hematology, clinical chemistry, urinalysis), vital signs, ECG parameters, physical examination, blood pressure measurement, heart rate, pulmonary function test, and Visual Analog Sedation Scale (VASS) over the course of the study. AEs were documented. For PK testing, venous blood samples were collected to measure the concentrations of ENA-001 in K2EDTA human plasma at various points in time over 24 hours, beginning with the commencement of the IV infusion. The PK parameters of interest were Cmax, area under the curve (AUC) (tf, 0-24, 0-∞, 0-∞/D), Tmax, t½, CLp, Vz, and Vss. PD evaluations were minute ventilation, tidal volume, respiratory rate on the Dräger scale, and tidal volume and respiratory rate on the NOX/T3 instrument, from which minute ventilation was calculated, using the formula of respiratory rate times the tidal volume. End-tidal CO_2_ and transcutaneous hemoglobin oxygen saturation (SpO_2_) were likewise assessed. Actigraphy was used to assess the quality of the respiratory recordings.

VivoSense® software (VivoSense, Newport Coast, California, USA) was customized and used to import, analyze, and report the Dräger data and clinical event log files. Data were reviewed by a blinded polysomnographic technician to adjudicate quality scores and mark artifacts from predetermined criteria. PK analysis was performed using the WinNonlin Professional Edition (Version 5.3, Pharsight Corporation, Mountain View, California, USA), and all PK parameters and their related descriptive statistics were calculated using the WinNonlin software and Microsoft Excel (Version 2010). All PK parameters except for Tmax and t½ were logarithmically transformed and analyzed using the WinNonlin or Microsoft Excel programs. Geometric means, arithmetic means, standard deviations, and coefficients of variation were provided.

## Results

A total of 18 healthy adults were randomized into the study, of whom 17 completed the study. No replacement participants were used. There were 16 men and two women in the study, with a mean age of 35.6 years (range = 19 to 43 years). All participants were non-Hispanic Caucasians. The mean body mass index was 25.0 kg/m^2^ (range = 21.4 to 28.8). Pulmonary function tests at screening resulted in a mean percentage predicted forced expiratory volume in one second (FEV1) of 99.8% (range = 85% to 130%) and forced vital capacity (FVC) of 4.95 L (range = 3.14 to 6.59 L).

A total of 17 participants received a single IV infusion over two hours on the first day of each of the two treatment periods for a total of two doses per subject. One subject received only one infusion, which was a placebo; the subject withdrew consent for personal reasons after the first dose and reported a mild headache as an AE. Three IV doses were administered at a fixed-rate infusion of 0.96, 1.44, and 1.92 mg/kg/hour for two hours. During the third treatment period, two participants experienced increasing ventilatory efforts and decreasing ETCO_2_ levels with nadirs of 22 and 29 mmHg, respectively. Their infusions were stopped, resulting in a rapid diminution of ventilatory efforts. Therefore, it was considered that the stopping criteria for further dose escalation in these two participants had been achieved. During the fourth and final treatment period, participants were re-assigned to the two lower dose levels based on their previous treatments to increase the total number of participants receiving these dose levels. Over the course of the study, 78% of all participants (n = 14) reported at least one AE, of which the most common were vomiting, nausea, infusion-related reactions, headache, abdominal pain, diarrhea, dizziness, and hyperventilation. All AEs were deemed mild to moderate in severity, and no serious AEs or deaths occurred. AEs that were reported more than once are summarized in Table 1. There was no evidence of local or venous injury post-infusion, and infusion-site reactions or burning sensations occurred mainly at the two highest doses.

**Table 1:** Adverse events observed in >1 subject. Note that the doses of 0.96 and 1.44 mg/kg/hour were administered twice in the first and fourth and second and fourth treatment periods, respectively, while the highest doses was administered only once in the third period. NA: not applicable; AE: adverse event; IV: intravenous

Study period	1 / 4	2 / 4	3	All periods	Total participants reporting an AE
IV infusion rate (mg/kg/hour)	0.96	1.44	1.92	Placebo	
Number of participants total (n)	9	8	6	12	
Abdominal pain (n)	NA	2	NA	NA	2
Dizziness (n)	1	2	NA	NA	2
Headache (n)	1	1	NA	2	4
Hyperventilation (n)	NA	NA	2	NA	2
Loose stools (n)	NA	2	NA	NA	2
Nausea (n)	NA	4	5	NA	9
Venous pain, infusion site (n)	1	4	3	NA	8
Vomiting (n)	3	7	5	3	14
No. of participants with any AE (n)	3	7	5	3	14

Two participants terminated the high-dose (1.92 mg/kg/hour) ENA-001 infusion early and their AEs are summarized in Table 2.

**Table 2:** Summary of participants (107 and 109) whose adverse events led to early discontinuation of treatment. AE: adverse event

Subject	Dose mg/kg/hour	Start	Stop	AE Severity	Time of AE		Duration of AE	Time	ng/mL
					Onset	End			
107	1.92	9:15	10:11	Moderate HV	9:55	10:11	16 minutes	9:45	914.7
								10:00	1200
				Mild nausea	10:00	11:30	30 minutes	10:15	1209
								10:45	651
109	1.92	9:25	10:46	Moderate HV	10:45	11:15	30 minutes	10:27	2022
								10:55	1565
								11:23	944.9
				Moderate vomiting	10:45	10:48	3 minutes	10:55	1565
								11:23	944.9

Consistent with the mechanism of action of ENA-001, hyperventilation occurred from 40 to 80 minutes after infusion commenced and was associated with plasma concentrations of 1,200 to 2,000 ng/mL. The same two participants experienced hyperventilatory events summarized in Table 3. The mean individual PK parameters in volunteers at the three infusion rates are summarized in Table 4.

**Table 3:** Pharmacodynamic and safety assessment associated with hyperventilation. Subject 107 presented with typical hyperventilation parameters, including a 70% increase in MV drive by tidal volume and a 20% decrease in ETCO2. Subject 109 had an approximately 25% decrease in ETCO2. Neither hyperventilating subject exhibited substantial cardiovascular symptoms in response to the increase in ventilation. Signs and symptoms in both patients resolved rapidly as soon as the treatment was discontinued and plasma levels of the study drug decreased. BP: blood pressure; bpm: breaths per minute; BPM: beats per minute; ETCO2: end-tidal carbon dioxide; MV: minute ventilation; RR: respiratory rate; SpO_2_: oxygen saturation; TV: tidal volume

	Subject 107			Subject 109		
	Baseline (time)	Onset	Resolution	Baseline	Onset	Resolution
	8:30	9:55	10:15	8:40	10:45	11:15
ETCO_2_ (%)	33.63%	26.7%	29.10%	34.83%	26%	34.5%
MV (L/minute)	8.51	14.54	8.99	5.88	8.38	6.71
RR (bpm)	14.37	16.7	13.6	10.67	12.88	11.9
TV (mL)	570	828	789	545	584	539
SpO_2_ (%)	97%	99%	99%	97%	99%	97%
Heart rate (BPM)	53.4	63.10	57.30	77	85.9	78.1
BP (mmHg)	123/79	124/86	125/79	121/83	126/88	119/82

**Table 4:** Mean values of pharmacokinetic parameters in individuals at three infusion rates. All rates are mg/kg/minute over a period of two hours. AUC: area under the curve of a plasma concentration versus the time profile; CLp: total plasma clearance; Cmax: maximum concentration; T-1/2: elimination half-life; Tmax: time to reach Cmax; VSS: volume of distribution at steady stage; VZ: volume of distribution elimination

Infusion rate ± SD	T-1/2 (h)	Tmax (h)	Cmax (ng/mL)	AUC0-24 (Ng×h/mL)	AUC0-∞ (mg/kg)	AUC0-∞/D (mg×h/mL)	Vz (L/kg)	VSS (L/kg)	CLp (mL/minute/kg)
0.016 mg/kg/minute	6.97	1.87	882	2,669	2,759	1,438	7.19	2.57	12.3
SD	1.40	0.20	201	648	677	353	1.55	0.80	3.4
0.024 mg/kg/minute	5.24	1.92	1347	3,916	3,969	1,378	5.62	1.82	12.7
SD	0.98	0.17	299	880	915	319	1.04	0.29	3.0
0.032 mg/kg/minute	8.12	1.85	1,935	5,878	6,105	1,590	7.44	2.49	10.6
SD	0.53	0.24	276	799	839	217	0.77	0.36	1.63

There were eight AEs of infusion-site pain, all deemed mild to moderate in severity. The onset of infusion-site pain occurred with the commencement of the infusion for 5/8 participants and ended when the infusion was discontinued in 4/8 participants. No venous irritation was observed.

**Figure 1:**
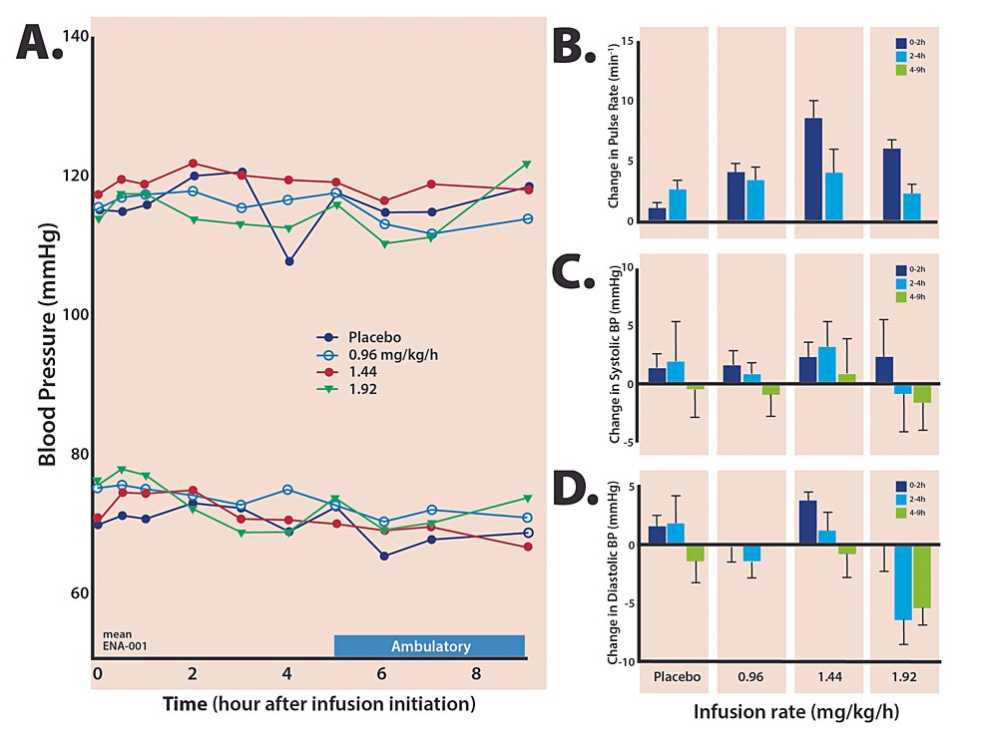
Mean systolic and diastolic blood pressure values after commencement of the injection appear in panel A. Changes from the pre-dose were calculated and integrated over the two-hour infusion, two hours after discontinuation of infusion, and to the end of the assessment period at nine hours. Panels B, C, and D show pulse rates, systolic, and diastolic blood pressure values, respectively.

No abnormalities in clinical laboratory values were observed. Blood pressure was assessed at predetermined time points across the study (Figure 1). There were no significant changes in diastolic blood pressure with ENA-001 versus placebo during the infusion. At the low dose (0.96 mg/kg/hour), there was no change in mean diastolic blood pressure (-0.2%) during the infusion; the middle dose (1.44 mg/kg/hour) showed an increase of 5.2% which did not differ significantly from placebo (+2.5%). At the highest dose (1.92 mg/kg/hour), diastolic blood pressure did not change (-0.2%) and was similar to the low dose. When the infusion was terminated, diastolic blood pressure decreased over the next two hours in all infusion groups, but it remained unchanged for placebo patients.

Systolic blood pressure exhibited no significant changes versus placebo during the course of the infusion.

Using the continuous ECG monitoring data, pulse rates were derived by extracting them from the 30-minute window before the infusion and then over the next four hours once the infusion started. No unexpected changes or outliers in pulse rate data were observed. Among all groups treated with ENA-001, the mean pulse increase ranged from 4.1 to 8.7 beats per minute (bpm), which can be compared to the placebo periods, whose pulse rate increased by 2.8 bpm after infusion. The maximum heart rate observed in an individual was 91.5 bpm in a subject whose baseline rate was 70 bpm and who received a dose of 1.44 mg/kg/hour. Serial 12-lead ECG was performed on Day 1 of the treatment continuously from the start of infusion for four hours. No clinically important changes were observed for the QT interval, RR interval, QTc, QTcB, or QTcF.

Pulmonary function was tested at screening, at baseline (Day 1) and on the first day of treatment approximately 0.25 hours after the infusion was stopped. No clinically important changes in pulmonary function assessment were observed at any of the doses from screening to the termination of the study.

Sedation was tested using a 100 mm visual analog scale, where 1 meant fully awake and 100 meant almost asleep. After a pre-dose assessment, participants self-graded their sedation levels every 30 minutes from the start of the infusion over the next four hours. If a subject fell asleep, the maximum score of 100 mm was assigned for that time interval. Thus, decreases from baseline indicate increased alertness, while increases indicate greater sedation. Using group means for the dose groups, there were no significant differences for the 0.96 and 1.44 mg/kg/hour groups compared to placebo, but sedation increased in the 1.96 mg/kg/hour group versus placebo (Table 5).

**Table 5:** Using a visual analog scale where 1 was full wakefulness and 100 was nearly or fully asleep, participants were evaluated at baseline before dosing and a group mean established. Subsequent values in the table show changes against the group mean at the measurement intervals. Note that negative numbers indicate increased alertness.

Time following the start of infusion in hours	Placebo (n = 12)	0.96 mg/kg/hour (n = 9)	1.44 mg/kg/hour (n = 8)	1.96 mg/kg/hour (n = 6)
Baseline (group mean)	50.7	56.3	65.5	37.5
0.5	-6.4	5.3	-2.4	5.2
1.0	-5.9	5.2	-8.1	10.7
1.5	6.7	-6.7	-10.4	20.6
2	-15.2	-20.6	-28.1	8.8
2.5	-18.8	-31.3	-16.8	5.8
3	-18.6	-16.3	-8.1	8.3
4	-28.6	-29.7	-20.5	-16.7

The Columbia-Suicide Severity Rating Scale was used to measure the suicidal ideation of participants before entering the study. All participants had a score of 0, indicating no suicidal ideation or suicidal behavior. At the follow-up visit, these scores were unchanged.

Pharmacodynamics

**Figure 2:**
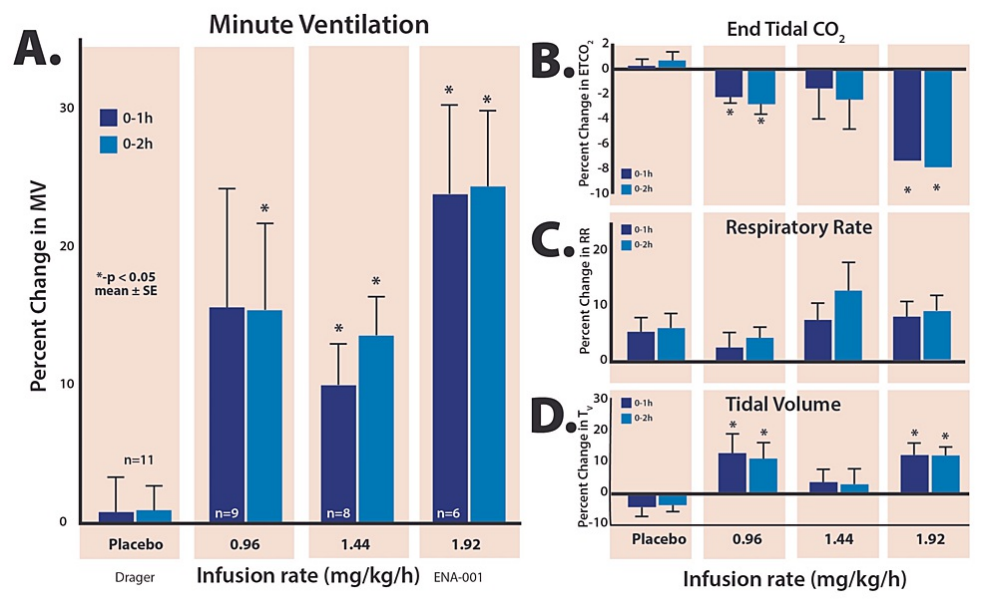
The integrated percent change from baseline during the first hour (0-1 hours) and over the course of the first two hours (0-2 hours) after starting the infusion for minute ventilation (panel A), endtidal CO2 (panel B), respiratory rate (panel C), and tidal volume (panel D).

For the first dose of ENA-001 (0.96 mg/kg/hour over two hours), VE, as measured by the pneumotachometer increased rapidly, with a mean 15.4% increase during a two-hour infusion period. The middle dose had a similar increase at 13.6% while the high dose exhibited an increase of 24.3%. VE changes were statistically significant at all three dose levels (Figure 2). Note that ETCO_2_ was reduced at all dose levels; the highest dose was associated with a >8% reduction over the course of the infusion.

**Figure 3:**
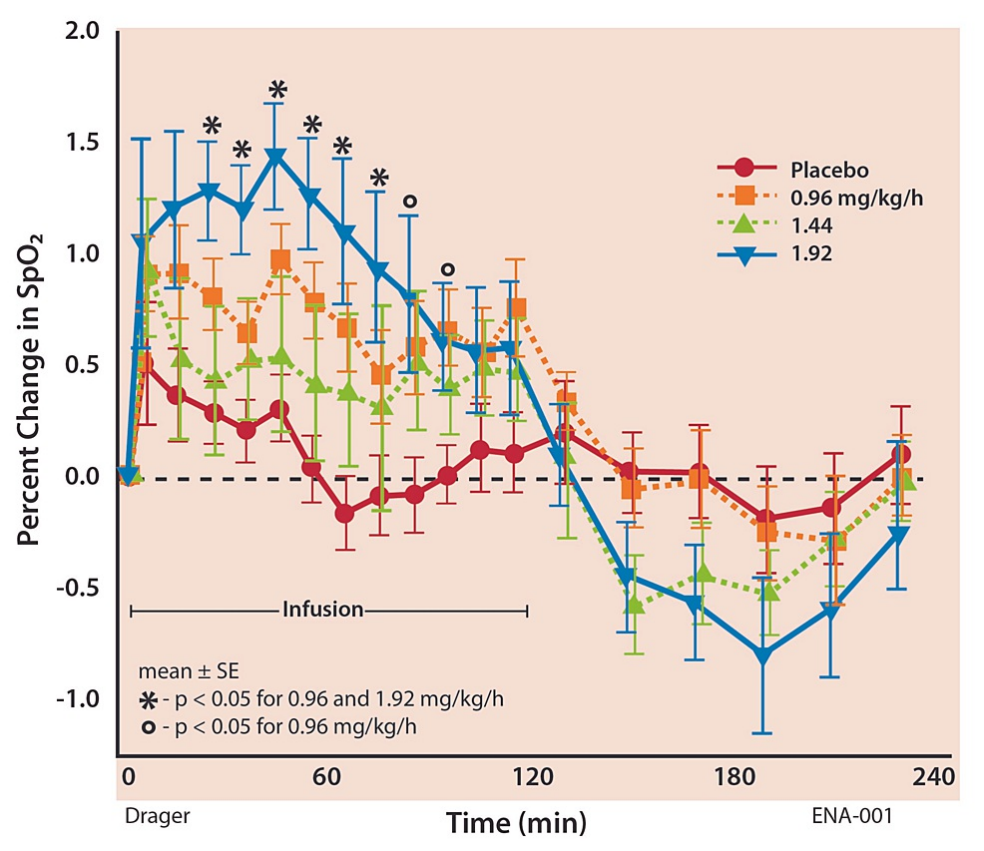
Transcutaneous hemoglobin oxygen saturation (Dräger scale).

Hemoglobin oxygen saturation (SpO_2_) increased rapidly once infusion started at the dose of 0.96 mg/kg/hour and there were statistically significant differences from 25 to 75 minutes for low and high doses, respectively (Figure 3). Integrated effects over the two-hour infusion period increased 0.70% and 0.97% (EG1) for low and high doses, respectively, compared to placebo (0.13%, p < 0.05). For the mid-range dose, the one-to-two-hour period had a greater increase in minute ventilation (17.2%) than the first hour (10.0%) and showed a statistically significant increase in SpO_2_ (0.41% vs. -0.02%, p < 0.05).

Pharmacokinetics

**Figure 4:**
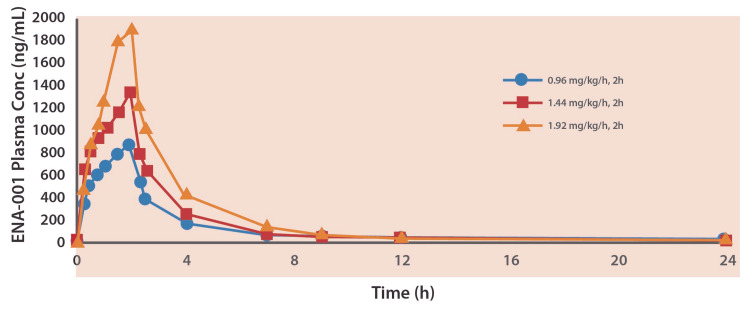
The mean plasma concentration-time profiles of ENA-001 during and following a two-hour intravenous infusion in healthy participants.

Plasma concentration-time profiles were similar among all doses and groups, showing that plasma level rose rapidly during the infusion and fell sharply at first, then more gradually over the course of the infusion, as shown in Figure 4.

Mean Cmax values ranged from 860 to 1,919 ng/mL and generally were achieved toward the end of the two-hour infusion period. Once the infusion terminated, all plasma concentration-time profiles displayed apparent bi-exponential decay with a steep distributional phase and a gradual terminal phase. The mean terminal half-life of the agent ranged from 5.2 to 8.1 hours. The PK parameters of ENA-001 in healthy participants could be described as a two-compartment model with a distributional half-life of under 20 minutes.

Mean systemic plasma clearance values (CLp) ranged from 10.5 to 12.4 mL/minute/kg and were approximately half of the hepatic blood flow rate in humans. The mean steady-state volume of distribution values (Vss) ranged from 1.8 to 2.5 L/kg and was approximately two to three times the total body water in humans, which suggests that ENA-001 is highly distributed throughout bodily tissues. Intersubject variability in PK parameters was no greater than 20% for t½, 28% for CLp, and 31% for VSS.

## Discussion

ENA-001 had reliably predictable PK behavior in healthy volunteers when administered within the dose ranges of 0.96 to 1.92 mg/kg/hour as a continuous IV infusion over two hours. Plasma concentrations rose rapidly during infusion, then when infusion terminated, initially fell sharply and then more gradually. This PK profile could be described as a “fast on, fast off” product profile and suggests that the drug’s pharmacological effects are driven by the Cmax more than the AUC-drive model observed in animal studies. A two-compartment model with a distribution half-life under 30 minutes was observed. Both Cmax and AUC 0-∞ are approximately dose-proportional. The values for t½, CLp, and VSS seem to be dose-independent. These results suggest that dose regimens for therapeutic use could be fairly straightforward.

The maximum plasma concentration achieved during this study was 2,150 ng/mL, which was attained at the end of the two-hour infusion at the highest dose. The highest total systemic exposure was 6,450 ng × hour/mL, likewise achieved at the highest dose. Preclinical limits for maximum plasma concentration and total systemic exposure had been set for this study at Cmax of 2,200 ng/mL and AUC0-∞ at 7900 ng × hour/mL. These limits were not exceeded.

The terminal half-life expressed as the geometric mean of t½ was 6.33 hours. This suggests little drug accumulation in the body after 22 hours, which aligns with the finding that there were low measurable plasma concentrations of the drug 24 hours after the start of the infusion. Systemic plasma clearance was moderate to high at 11.8 mL/minute/kg, which is approximately half of the hepatic blood flow rate in humans. The volume of distribution appears to be high (the geometric mean of the Vss is 2.19 L/kg).

Thus, the PK of ENA-001 can be considered appropriate for continuous IV infusion over the tested duration of two hours in healthy humans at the tested dose ranges from 0.96 to 1.92 mg/kg/hour. ENA-001 stimulates minute ventilation at the lowest infusion rate of 0.96 mg/kg/hour without blood pressure responses of similar magnitude. ETCO_2_ decreased with ENA-001. At the highest dose of 1.92 mg/kg/hour, ENA-001 was associated with dose-limiting AEs of nausea and vomiting.

The role of a respiratory stimulant may have significant clinical applications. It may be able to drive respiration following surgery without the necessity of using naloxone, which can reverse analgesia [14]. A recent proof-of-concept study showed that a respiratory stimulant may be a treatment option for apnea of prematurity, a condition affecting preterm neonates [15]. As new guidance emerges for respiratory distress syndrome, respiratory stimulants may play an important role in treatment [16]. Death caused by an overdose of illicit drugs, particularly opioids, is the primary cause of death for people between the ages of 25 and 64 [17], exacerbated by an increase in synthetic street drugs, including illicit fentanyl [18,19]. Many street drugs are adulterated, and with an increase in polysubstance abuse, emergency medical personnel often have to treat patients in respiratory distress without knowing which drugs they have taken [20]. As a reversal agent, naloxone has limitations [21]. A respiratory stimulant may play an important role in resuscitation from overdose.

There are several other respiratory stimulants, including caffeine, almitrine, and doxapram. Caffeine is primarily used to treat apnea of prematurity but has been associated with cardiovascular adverse effects [22]. Caffeine is analeptic and stimulates respiration by way of inhibiting adenosine receptors and/or phosphodiesterase at the brainstem level [23]. Almitrine is associated with peripheral neuropathy which limits its use [24]. The best-known agent of these three is doxapram. Doxapram has been on the market for over 50 years, but it has been used mainly in the niche populations of neonatal respiratory care, postanesthesia respiratory care, and intensive care [22]. In a systematic review and meta-analysis, doxapram was evaluated in four clinical trials (n = 176) and was modestly superior to placebo in avoiding blood-gas deterioration over the first few hours of treatment [25]. Doxapram has been associated with cardiovascular adverse events and central nervous system stimulatory effects that limit its use [22,25].

This study has several limitations. It was a single-site study with a small population. The study was designed to measure important PK and PD values but was not powered to demonstrate the efficacy of the drug. As we addressed PK parameters in a study population of healthy individuals only, these results may not be generalizable to populations with, for example, comorbid conditions or respiratory problems, which may be more reflective of real-world clinical practice.

## Conclusions

ENA-001 is a novel, first-in-class respiratory stimulant that showed reliably predictable PK and PD properties in an ascending-dose study in healthy volunteers. Doses were two-hour continuous infusions of 0.96, 1.44, and 1.96 mg/kg/hour. Ventilation increases from ENA-001 were not accompanied by similar-magnitude increases in blood pressure. ENA-001 was associated with decreases in ETCO_2_ and concomitant small albeit statistically significant increases in SpO_2_ levels. ENA-001 exhibited reliable PK and PD characteristics and was safe, effective, and well-tolerated in healthy participants.
